# Influence of socioeconomic and demographic status on spirometry testing in patients initiating medication targeting obstructive lung disease: a population-based cohort study

**DOI:** 10.1186/1471-2458-13-580

**Published:** 2013-06-14

**Authors:** Mette M Koefoed, Jens Søndergaard, René dePont Christensen, Dorte E Jarbøl

**Affiliations:** 1 Research Unit of General Practice, Institute of Public Health, University of Southern Denmark, J.B. Winsløws Vej 9A, 1, DK-5000, Odense C, Denmark

**Keywords:** Socioeconomic Status, Spirometry, Obstructive Lung Disease

## Abstract

**Background:**

Socioeconomic status is known to influence the prevalence, severity and mortality of obstructive lung diseases, but it is uncertain whether it affects the use of diagnostic spirometry in patients initiating treatment for these conditions. The objective of this paper was to examine a possible association between education, income, labour market affiliation, cohabitation status and having spirometry performed when initiating medication targeting obstructive pulmonary disease.

**Methods:**

We conducted a population-based cohort study. Danish national registers were linked, retrieving data on prescriptions, spirometry testing, socioeconomic and demographic variables in all first time users of medication targeting obstructive lung disease in 2008.

**Results:**

A total of 37,734 persons were included and approximately half of the cohort had spirometry performed. Among medication users under 65 years of age, being unemployed was significantly associated with reduced odds of having spirometry performed, the strongest association was seen in men (OR = 0.82, CI = 0.73-0.91). Medium income was associated with increased odds of having spirometry performed in men (OR = 1.18, CI = 1.06-1.30) and high educational level (>12 years) was associated with reduced odds of having spirometry performed in women (OR = 0.86, CI = 0.78-0.94). Cohabitation status was not associated with having spirometry performed. Among medication users over 65 years of age, living alone was associated with reduced odds of having spirometry performed among men (OR = 0.78, CI = 0.69-0.88).

**Conclusion:**

Social inequity in spirometry testing among patients initiating medication targeting obstructive lung disease was confirmed in this study. Increased focus on spirometry testing among elderly men living alone, among the unemployed and among women with higher education is required when initiating medication.

## Background

Low socioeconomic status is associated with increased prevalence and higher severity of chronic bronchitis [1], asthma [2] and chronic obstructive pulmonary disease (COPD) [3]. Studies have demonstrated poorer quality of life [4], poorer controlled asthma [5], lower lung function, increased risk of hospitalisation [6,7], and a higher mortality rate [8,9] among obstructive lung disease patients with low socioeconomic status. Socioeconomic inequalities in health are well known, but despite free access to health care, socioeconomic status is also found to be associated with disease management. Specifically, this is shown in neurological and cardiovascular illnesses where studies have demonstrated an unequal use of diagnostic tests like computer tomography (CT) and magnetic resonance imaging (MRI) in stroke patients, and angiography in patients with acute myocardial infarction [10-12].

Spirometry is considered the gold standard for diagnosing asthma and COPD [13,14]. Despite recommendations, spirometry is underused in patients being diagnosed with these illnesses [15,16]. In a previous study we found a lack of spirometry use when patients initiated medication targeting obstructive lung disease [17], but it is uncertain whether underuse of spirometry is due to social inequalities in diagnostic testing. The purpose of this study was therefore to assess, whether there is an association between socioeconomic and demographic factors like education, income, affiliation to the labour market, cohabitation status and having spirometry performed when initiating medication targeting obstructive lung disease.

## Methods

A register-based cohort study covering the entire population of Denmark with currently 5.5 million inhabitants was performed. The healthcare system in Denmark is tax funded, thereby giving all inhabitants, irrespective of socioeconomic status, free access to all services in general practice and hospital care, including spirometry [18].

All Danish citizens are registered in the Danish Civil Registration System and assigned a unique civil registration number, which is used in all national registers, enabling accurate linkage between them [19,20]. The national registers used in this study are all maintained and stored in Statistics Denmark, where researchers can apply for access to these data.

This project is register-based and according to “The Act on Research Ethics Review of Health Research Projects in Denmark” only questionnaire surveys and medical database research projects involving human biological material are required to be notified to the research ethics committee. The research ethics committee has, therefore, not been contacted. The study was approved by the Danish Data Protection Agency, J.nr. 2011-41-5798.

### Study subjects

Patients were identified in the National Prescription Register, a register containing complete information on all redeemed prescriptions since 1997. We identified all adults who were first time users of medication targeting obstructive lung disease in 2008. Firstly, all patients redeeming drugs targeting obstructive lung disease, defined as the anatomical therapeutic chemical (ATC) code R03, in 2008 were identified. We then excluded patients who were either under 18 years of age on 1 January 2008 or who had previous records of prescriptions with ATC code R03 in the register (1995–2007). We divided ATC code R03 medication into three main categories: beta-2-agonists, anticholinergics and inhaled corticosteroids. Other medications within the ATC R03 category were rarely prescribed and therefore excluded from categorisation. Further, we examined whether patients had repeated redemption of pulmonary medication exceeding one month. We defined patients as having “high severity” of respiratory illness if they initiated two or more medication categories within the first year and had repeated redemption of pulmonary medication.

### Health care utilisation - spirometry when initiating medication

All spirometric procedures registered in the National Health Service Register and the National Patient Registry between 2007–2010 were extracted. These two registries contain information on all services provided in the healthcare system. For each patient we assessed if spirometry was registered in an 18-month period from 6 months before to 12 months after the date of redeeming the first prescription of obstructive lung medication.

As a prerequisite for reimbursement of spirometry, general practice is obliged to be enrolled in a quality improvement programme ensuring calibration of spirometers and other diagnostic equipment on a regular basis. Also, a full spirometry with three measurements is recommended, including recording of both fev1 and FVC to qualify for reimbursement. The registries do not contain data on the results of these measurements. Peak flow measurements are coded separately and are not included in our analysis.

### Socioeconomic variables

The following socioeconomic and demographic variables were included: education, income, affiliation to the labour market and cohabitation status [12,21]. In order for patients to be included in the study, all variables had to be available. Highest attained educational level in 2008 was extracted and divided into three categories: <10 years, (primary and lower secondary), 10–12 years (vocational training and upper secondary school) and >12 years (higher education). Average disposable income the previous 5 years (2003–2007) was extracted and defined as the entire household income after taxation, adjusted for number of persons in the household. Disposable income was categorised as low (first quartile), medium (second and third quartile) or high (fourth quartile). Affiliation to the labour market was extracted and divided into three categories: working, retired or unemployed, according to the status each individual predominantly had in 2008. Cohabitation status in 2008 was categorised as married/cohabiting or living alone (divorced, widowed or never married).

### Statistical analysis

All analyses were stratified into two age groups: <65 years and ≥65 years. This is the normal retirement age in Denmark and we found stratified analyses appropriate as there was a substantial difference in labour market status, income and education between these two groups. Analyses were both conducted overall and stratified according to gender, because studies have demonstrated that gender can modify the effect of socioeconomic factors [22]. Logistic regression models were used to calculate crude and adjusted odds ratios (ORs) with 95% CIs for the associations between socioeconomic variables and having spirometry performed in the defined 18-month period. Confounders adjusted for were gender, age and severity of illness. P-values < 0.05 were considered statistically significant. All statistical analyses were carried out using STATA 11 (STATACorp, College Station, TX, USA).

## Results

A total of 37,734 persons fulfilled the inclusion criteria. Mean age of the study cohort was 52.5 years, 46.7% were male. Baseline characteristics are shown in Table 1. Approximately half of the cohort (19,119 (50.7%)) had spirometry performed during the 18-month time interval (Table 2).

**Table 1:** Socioeconomic and demographic characteristics of study cohort

**Baseline characteristics**	**All**	**< 65 years**		**≥65 years**	
		**Men**	**Women**	**Men**	**Women**
	**n = 37734**	**n = 12245**	**n = 14464**	**n = 5391**	**n = 5634**
Age, mean (SD)	52.5 (SD 18.1)	47.4 (SD 13.0)	45.8 (SD 13.5)	76.6 (SD 6.1)	77.1 (SD 6.4)
High severity (%)	9903 (26.2)	2776 (22.7)	3040 (21.0)	2106 (39.0)	1981 (35.1)
Highest attained education (%)					
<10 years	13916 (36.9)	3765 (30.7)	4428 (30.6)	2358 (43.7)	3365 (59.7)
10-12 years	15727 (41.7)	5774 (47.2)	6127 (42.4)	2231 (41.4)	1595 (28.3)
>12 years	8091 (21.4)	2706 (22.1)	3909 (27.0)	802 (14.9)	674 (12.0)
Income (%)					
Low (1st quartile)	9433 (25.0)	2257 (18.4)	2867 (19.8)	1852 (34.4)	2457 (43.6)
Medium (2nd + 3rd quartile)	18868 (50.0)	6388 (52.2)	7458 (51.6)	2558 (47.4)	2464 (43.7)
High (4th quartile)	9433 (25.0)	3600 (29.4)	4139 (28.6)	981 (18.2)	713 (12.7)
Labour market status (%)					
Working	21374 (56.6)	9774 (79.8)	11060 (76.5)	389 (7.3)	151 (2.7)
Retirement pension	11587 (30.7)	524 (4.3)	699 (4.8)	4943 (91.7)	5421 (96.2)
Unemployed	4773 (12.7)	1947 (15.9)	2705 (18.7)	59 (1.0)	62 (1.1)
Cohabitation (%)					
Married/cohabiting	24049 (63.7)	8252 (67.4)	9702 (67.1)	3698 (68.6)	2397 (42.5)
Living alone	13685 (36.3)	3993 (32.6)	4762 (32.9)	1693 (31.4)	3237 (57.5)

**Table 2:** Proportion of patients receiving spirometry in the 18-month time period by socioeconomic status

	**All ages**			**<65 years**			**≥65 years**		
	**Men**	**Women**	**All**	**Men**	**Women**	**All**	**Men**	**Women**	**All**
n (%)	9443 (53.5)	9676 (48.1)	19119 (50.7)	6336 (51.7)	6792 (47.0)	13128 (49.2)	3107 (57.6)	2884 (51.2)	5991 (54.3)
Highest attained education n (%)									
<10	3291 (53.7)	3839 (49.3)	7130 (51.2)	1963 (52.1)	2160 (48.8)	4123 (50.3)	1328 (56.3)	1679 (49.9)	3007 (52.5)
10-12	4376 (54.7)	3770 (48.8)	8146 (51.8)	3048 (52.8)	2921 (47.7)	5969 (50.2)	1328 (59.5)	849 (53.2)	2177 (56.9)
>12	1776 (50.6)	2067 (45.1)	3843 (47.5)	1325 (49.0)	1711 (43.8)	3036 (45.9)	451 (56.2)	356 (52.8)	807 (54.7)
Income n (%)									
Low (1st quartile)	2066 (51.5)	2430 (46.7)	4608 (48.8)	1099 (48.7)	1291 (45.0)	2390 (46.6)	1028 (55.5)	1190 (48.4)	2218 (51.5)
Medium (2nd + 3rd quartile)	4952 (54.5)	4862 (48.3)	9658 (51.2)	3347 (52.4)	3502 (47.0)	6849 (49.5)	1512 (59.1)	1297 (52.6)	2809 (55.9)
High (4th quartile)	2425 (53.5)	2384 (49.5)	4853 (51.4)	1890 (52.5)	1999 (48.3)	3889 (50.3)	567 (57.8)	397 (55.7)	964 (56.9)
Labour market status n (%)									
Working	5242 (51.6)	5170 (46.1)	10412 (48.7)	5008 (51.2)	5098 (46.1)	10106 (48.5)	234 (60.2)	72 (47.7)	306 (56.7)
Retirement pension	3177 (58.1)	3168 (51.8)	6345 (54.8)	335 (63.9)	389 (55.7)	724 (59.2)	2842 (57.5)	2779 (51.3)	5621 (54.2)
Unemployed	1024 (51.0)	1338 (48.4)	2362 (49.5)	993 (51.0)	1305 (48.2)	2298 (49.4)	31 (52.5)	33 (53.2)	64 (52.9)
Cohabitation n(%)									
Married/cohabiting	6457 (54.0)	5836 (48.2)	12293 (51.1)	4260 (51.6)	4534 (46.7)	8794 (49.0)	2197 (59.4)	1302 (54.3)	3499 (57.4)
Living alone	2986 (52.5)	3840 (48.0)	6826 (49.9)	2076 (52.0)	2258 (47.4)	4334 (49.5)	910 (53.8)	1582 (48.9)	2492 (50.5)

### Association between socioeconomic status and spirometry testing in medication users < 65 years

There was a significant association between affiliation to the labour market and having spirometry performed; being unemployed was significantly associated with a reduced chance of spirometry testing in both sexes, the strongest association was seen in men (OR = 0.82, CI = 0.73-0.91). Medium and high income was associated with an increased OR of having spirometry performed in men. However, only medium income was statistically significant (OR = 1.18, CI = 1.06-1.30). This association was not seen in women. High educational level (>12 years) was associated with a reduced chance of spirometry testing in the total group and among women (OR = 0.86, CI = 0.78-0.94). Cohabitation status was not associated with having spirometry performed in either sex (Table 3).

**Table 3:** Association between socioeconomic status and spirometry in patients < 65 years

**Under 65 years**	**Men**		**Women**		**All**	
	**Crude OR (95% CI)**	**Adjusted OR (95% CI)**	**Crude OR (95% CI)**	**Adjusted OR (95% CI)**	**Crude OR (95% CI)**	**Adjusted OR (95% CI)**
				**p-value**	**p-value**	**p-value**
Age (increasing)	1.01 (1.01-1.01)	-	1.01 (1.01-1.01)	-	1.01 (1.01-1.01)	-
	P < 0.001		P < 0.001		P < 0.001	
Gender	-	-	-	-	0.83 (0.79-0.87)	-
					P < 0.001	
High severity						
No	1	-	1	-	1	-
Yes	6.19 (5.57-6.88)	-	6.89 (6.25-7.60)	-	6.57 (6.11-7.06)	-
	P < 0.001		P < 0.001		P < 0.001	
Highest attained education						
<10	1	1	1	1	1	1
10-12	1.03 (0.95-1.11)	1.04 (0.95-1.13)	0.96 (0.89-1.03)	1.00 (0.92-1.08)	0.99 (94–1.05)	1.01(0.95-1.08)
	P = 0.534	P = 0.413	P = 0.262	P = 0.929	p = 0.815	p = 0.679
>12	0.88 (0.80-0.97)	0.92 (0.83-1.03)	0.82 (0.75-0.89)	0.86 (0.78-0.94)	0.84 (0.78-0.89)	0.88 (0.82-0.95)
	P = 0.012	P = 0.137	P < 0.001	P = 0.001	P < 0.001	P < 0.001
Income (quartiles)						
1st	1	1	1	1	1	1
2nd + 3rd	1.16 (1.05-1.28)	1.18 (1.06-1.30)	1.08 (0.99-1.18)	0.99 (0.90-1.09)	1.12 (1.05-1.19)	1.08 (1.00-1.13)
	P = 0.002	P = 0.002	P = 0.079	P = 0.882	p = 0.001	p = 0.039
4th	1.16 (1.05-1.29)	1.12 (1.00-1.26)	1.14 (1.04-1.25)	1.00 (0.89-1.11)	1.16 (1.08-1.24)	1.06 (0.98-1.14)
	P = 0.005	P = 0.052	P = 0.007	P = 0.981	P < 0.001	p = 0.177
Labour market status						
Working	1	1	1	1	1	1
Retirement pension	1.69 (1.41-2.02)	1.20 (0.98-1.48)	1.47 (1.26-1.71)	1.07 (0.89-1.27)	1.54 (1.37-1.73)	1.12 (0.98-1.28)
	P < 0.001	P = 0.082	P < 0.001	P = 0.475	P < 0.001	p = 0.091
Unemployed	0.99 (0.90-1.09)	0.82 (0.73-0.91)	1.09 (1.00-1.19)	0.91 (0.83-1.00)	1.04 (0.97-1.10)	0.87 (0.81-0.93)
	P = 0.849	P < 0.001	P = 0.045	P = 0.049	p = 0.272	P < 0.001
Cohabitation						
Married/Cohabiting	1	1	1	1	1	1
Living alone	1.01 (0.94-1.09)	0.99 (0.91-1.07)	1.03 (0.96-1.10)	1.03 (0.95-1.11)	1.02 (0.97-1.07)	1.01 (0.95-1.07)
	P = 0.703	P = 0.752	P = 0.438	P = 0.492	p = 0.423	p = 0.762

Adjusted for gender, age and severity.

### Association between socioeconomic status and spirometry testing in medication users ≥ 65 years

Living alone was associated with a reduced chance of having spirometry performed and this was statistically significant in the total group and among men (OR = 0.78, CI = 0.69-0.88), but not among women. Medium length education (10–12 years) and medium income were associated with increased OR of spirometry testing in the total group, but were not statistically significant in the gender stratified analysis. No association between labour market affiliation and having spirometry performed was shown, Table 4.

**Table 4:** Association between socioeconomic status and spirometry in patients ≥ 65 years

**Over 65 years**	**Men**		**Women**		**All**	
	**Crude OR (95% CI)**	**Adjusted OR (95% CI)**	**Crude OR (95% CI)**	**Adjusted OR (95% CI)**	**Crude OR (95% CI)**	**Adjusted OR (95% CI)**
					**(p-value)**	**(p-value)**
Age (increasing)	0.97 (0.96-0.98)	-	0.96 (0.95-0.97)	-	0.96 (0.96-0.97)	-
	P < 0.001		P < 0.001		P < 0.001	
Gender	-	-		-	0.77 (0.72-0.83)	-
					P < 0.001	
High severity						
No	1	-	1	-	1	-
Yes	3.65 (3.23-4.11)	-	4.09 (3.63-4.60)	-	3.89 (3.57-4.23)	-
	P < 0.001		P < 0.001		P < 0.001	
Highest attained education						
<10	1	1	1	1	1	1
10-12	1.14 (1.01-1.28)	1.09 (0.96-1.23)	1.14 (1.01-1.29)	1.10 (0.97-1.26)	1.19 (1.10-1.29)	1.10 (1.00-1.20)
	p = 0.028	p = 0.197	P = 0.028	P = 0.130	P < 0.001	p = 0.042
>12	1.00 (0.85-1.17)	0.98 (0.83-1.16)	1.12 (0.95-1.33)	1.13 (0.95-1.35)	1.09 (0.97-1.22)	1.05 (0.93-1.18)
	p = 0.967	p = 0.816	P = 0.166	P = 0.181	p = 0.143	p = 0.451
Income (quartiles)						
1st	1	1	1	1	1	1
2nd + 3rd	1.16 (1.03-1.31)	1.11 (0.98-1.27)	1.18 (1.06-1.32)	1.08 (0.95-1.22)	1.20 (1.10-1.30)	1.10 (1.00-1.20)
	p = 0.017	p = 0.113	P = 0.003	P = 0.241	P < 0.001	p = 0.047
4th	1.10 (0.94-1.28)	1.08 (0.91-1.29)	1.34 (1.13-1.58)	1.19 (0.99-1.42)	1.24(1.11-1.39)	1.12 (0.99-1.27)
	p = 0.242	p = 0.364	P = 0.001	P = 0.070	P < 0.001	p = 0.069
Labour market status						
Working	1	1	1	1	1	1
Retirement pension	0.90 (0.73-1.11)	1.01 (0.80-1.26)	1.15 (0.83-1.60)	1.40 (0.99-1.97)	0.91 (0.76-1.08)	1.13 (0.94-1.36)
	p = 0.307	p = 0.964	P = 0.386	P = 0.059	p = 0.269	p = 0.204
Unemployed	0.73 (0.42-1.27)	0.65 (0.37-1.16)	1.25 (0.69-2.26)	0.94 (0.50-1.75)	0.86 (0.58-1.28)	0.75 (0.49-1.13)
	p = 269	p = 0.146	P = 0.463	P = 0.841	p = 0.450	p = 0.169
Cohabitation						
Married/Cohabiting	1	1	1	1	1	1
Living alone	0.79 (0.71-0.89)	0.78 (0.69-0.88)	0.80 (0.72-0.89)	0.91 (0.81-1.02)	0.76 (0.70-0.82)	0.84 (0.77-0.91)
	P < 0.001	P < 0.001	P < 0.001	P = 0.119	P < 0.001	P < 0.001

Adjusted for gender, age and severity.

## Discussion

This study demonstrated that being unemployed reduced the odds of having spirometry performed among patients under 65 years of age. Furthermore, higher income was associated with increased odds of spirometry testing in men and high education reduced the odds of having spirometry performed among women. Among those aged 65 or above, medium income and medium length education increased the odds of spirometry in the total group, and living alone reduced the odds of spirometry testing in the total group and among men.

This study focused on the entire healthcare system, evaluating whether social inequity existed with regard to conducting spirometry in patients initiating treatment with medication for obstructive lung disease. In the Danish healthcare system general practitioners act as gatekeepers and healthcare coordinators for their patients, and they receive information on all contacts their patients have had to the rest of the healthcare system [18]. Coordination of follow-up is therefore an integral part of the GP’s work, and although the large majority of spirometry testing and prescribing is conducted by the general practitioners themselves, a diagnostic clarification of patients receiving prescriptions from other healthcare settings should be feasible within the defined 18-month frame. As this study is register-based, it enables us to include the entire population through wide-ranging administrative registries and link data on healthcare utilisation, socioeconomic and demographic status to each citizen. Although these national registries are comprehensive, some limitations must be kept in mind. The prescription database is complete for all redeemed prescriptions, thereby including all medication for obstructive lung disease, but patients who do not redeem prescribed medication will be misclassified in the register and therefore not be included in the cohort. However, we consider that this misclassification is insignificant as primary non-compliance is considered small [23]. All spirometry measures given in primary and secondary health care are assessed through two large administrative registries and an underreporting to these registers would lead to an underestimation of spirometry testing. Registering spirometry is a prerequisite for reimbursement, and registration is therefore assumed to be high, and underreporting is probably not a noteworthy problem. However, the registries contain no data on how the spirometry was conducted, and we cannot exclude some variation in the quality of these measurements. We required all socioeconomic and demographic variables to be present in the patients, and this criterion resulted in exclusion of 7.3% of the patients initiating medication in 2008. A majority of the patients excluded were either in the oldest age categories (>90 years) or immigrants, as they had no registered education. This underreporting to the registries is well known, and it is worth mentioning that immigrants and people over 90 years of age may be underrepresented in our cohort.

To the best of our knowledge, this is the first study assessing whether socioeconomic and demographic status influences spirometry testing in patients initiating obstructive pulmonary medication. Studies have demonstrated that low socioeconomic status is associated with fewer diagnostic tests in other illnesses [24-26], but these studies have only focused on acute illnesses in secondary care. Few studies from primary care have examined inequality in chronic disease management; Ashworth et al. [27] examined the association between social deprivation and having blood pressure monitored and found a lower proportion of patients having an updated blood monitoring in the most deprived residential areas compared to less deprived areas. Smith et al. [28], studied spirometry testing in chronic obstructive pulmonary disease management and found no socioeconomic gradient in different residential areas. Both these studies focused on monitoring procedures, not diagnostic testing, and they only reported aggregated data on socioeconomic status. A single study examined the influence of socioeconomic status on spirometry testing in the diagnostic process of asthma in Canada [16] and a significant association was found; higher income increased the likelihood of spirometry testing. This is in concordance with our findings among men under 65 years of age. In contrast, we found no influence of income on spirometry testing among women. A more pronounced influence of socioeconomic status among men has also been demonstrated in other studies [10].

Higher educational level did not increase the odds of spirometry testing as hypothesised; on the contrary, the opposite was seen in women less than 65 years of age. A similar opposing finding was demonstrated in a study of management of myocardial infarction in Denmark*.* High income increased the use of a procedure, but over time, high education decreased the use of the same procedure [12]. This demonstrates the fact that the effects of education and income can have opposite directions. There is no clear reason why high educational level reduces the odds of spirometry testing. One hypothesis could be that women with education and careers are too busy and not interested in a time-consuming diagnostic process despite it being free of charge. Patients who seem uninterested in further diagnostic examination may not have spirometry offered or they may decline coming to follow-up consultations [29].

Among men over 65 years we found a reduced chance of spirometry if they lived alone. Our findings are in concordance with other studies; being married/cohabitating has been associated with improved blood pressure control among the elderly [30]. Having a spouse is also shown to improve management of diabetes, primarily due to the positive influence a spouse has on health behaviour, and men seem more receptive to this positive influence [31].

Our study demonstrated that being unemployed was associated with not having spirometry performed. We found no other studies examining social inequity in disease management using this parameter. Studies have confirmed that unemployment has a great impact on health and mortality and this pattern is more pronounced among men [32-34]. These studies advocate two main hypotheses: one hypothesis is that unemployment is caused by pre-existing ill health, another that unemployment leads to adverse changes in health behaviour. Despite the fact that we adjusted for disease severity when medication was initiated, thereby adjusting for pre-existing respiratory illness, we still found a clear underuse of spirometry among the unemployed. The reason for this remains unanswered, but one explanation could be patients’ adverse health behaviour; they may also decline spirometry testing because they have fewer resources to engage in the diagnostic process.

## Conclusion

Spirometry is a prerequisite in all patients with suspected respiratory illness and should be performed to confirm a diagnosis in all patients receiving medication for these diseases. We have confirmed socioeconomic and demographic inequity in spirometry testing when patients initiate obstructive pulmonary medication. Increased focus on spirometry testing among elderly men living alone, among the unemployed and among women with higher education is required when initiating medication.

## Competing interests

The authors declare that they have no competing interests.

## Authors’ contributions

MK participated in the design of the study, performed statistical analysis and drafted the manuscript. RC participated in the design of the study and helped perform the statistical analysis. JS and DJ participated in the design of the study and helped draft the manuscript. All authors read and approved the final manuscript.

## Pre-publication history

The pre-publication history for this paper can be accessed here:

http://www.biomedcentral.com/1471-2458/13/580/prepub
